# Capacity building for cancer prevention and early detection in the Ugandan primary healthcare facilities: Working toward reducing the unmet needs of cancer control services

**DOI:** 10.1002/cam4.3659

**Published:** 2020-12-14

**Authors:** Alfred Jatho, Noleb M. Mugisha, James Kafeero, George Holoya, Fred Okuku, Nixon Niyonzima, Jackson Orem

**Affiliations:** ^1^ National Cancer Center Graduate School of Cancer Science and Policy Goyang Republic of Korea; ^2^ Uganda Cancer Institute Kampala Uganda

**Keywords:** cancer prevention, capacity building, early detection of cancer, integration of cancer services, primary health care, unmet needs for cancer prevention and early detection

## Abstract

**Background:**

In 2018, approximately 60,000 Ugandans were estimated to be suffering from cancer. It was also reported that only 5% of cancer patients access cancer care and 77% present with late‐stage cancer coupled with low level of cancer health literacy in the population despite a wide coverage of primary healthcare facilities in Uganda. We aimed to contribute to reducing the unmet needs of cancer prevention and early detection services in Uganda through capacity building.

**Methods:**

In 2017, we conducted two national and six regional cancer control stakeholders’ consultative meetings. In 2017 and 2018, we trained district primary healthcare teams on cancer prevention and early detection. We also developed cancer information materials for health workers and communities and conducted a follow‐up after the training.

**Results:**

A total of 488 primary healthcare workers from 118 districts were trained. Forty‐six health workers in the pilot East‐central subregion were further trained in cervical, breast, and prostate cancer early detection (screening and early diagnosis) techniques. A total of 32,800 cancer information, education and communication materials; breast, cervical, prostate childhood and general cancer information booklets; health education guide, community cancer information flipcharts for village health teams and referral guidelines for suspected cancer were developed and distributed to 122 districts. Also, 16 public and private‐not‐for‐profit regional hospitals, and one training institution received these materials. Audiovisual clips on breast, cervical, and prostate cancer were developed for mass and social media dissemination. A follow‐up after six months to one year indicated that 75% of the districts had implemented at least one of the agreed actions proposed during the training.

**Conclusions:**

In Uganda, the unmet needs for cancer control services are enormous. However, building the capacity of primary healthcare workers to integrate prevention and early detection of cancer into primary health care based on low‐cost options for low‐income countries could contribute to reducing the unmet needs of cancer prevention and early detection in Uganda.

## Abstract 2

In Uganda, the unmet needs for cancer control services are enormous. We engaged district leaders and healthcare managers, developed cancer information materials, and trained the district PHC workers to integrate prevention and early detection of cancer into the primary healthcare system. This could contribute to reducing the unmet needs of cancer control services in Uganda.

## 1 INTRODUCTION

In 2018, approximately 60,000 Ugandans were estimated to be suffering from cancer^1^ and need specialized care yet only 5% have access to medical services^2^ in the only one comprehensive cancer treatment center‐Uganda Cancer Institute. Out of the few who have access to cancer management in hospitals, most present with advanced cancer. A breast cancer study conducted in Uganda revealed that 77% of the patients presented to cancer hospital with a late‐stage cancer,^3^ which is characterized with poor prognosis. Individuals from rural areas of Uganda are more likely to present with an advanced stage cancer.^3^

There are increasing trends in incidence of the most common types of cancer in Uganda, especially breast, cervical, Kaposi sarcoma, and prostate cancer.^1^ Concerted efforts to reduce exposure to the known and putative cancer risk factors at all level of healthcare delivery could reduce the incidence of the preventable types of cancer. Moreover, the proportion of cancer patients with late‐stage presentation to cancer hospital and the resultant poor prognosis could be down‐staged and attenuated through affordable early detection strategies. These could save life and improve quality of life of many cancer patients.

The late detection of cancer could in part be attributable to limited knowledge on the benefits of early detection, scarcity of cancer screening and diagnostic facilities within the reach of the communities, the inadequate cancer information and skills among primary healthcare workers,^3^, ^4^, ^5^ and prompt cancer management.^6^, ^7^ Scarcity of adequate health facilities with the skilled and facilitated capacity to provide the key cancer prevention information and conduct the screening and diagnostic procedures have been reported.^8^, ^9^, ^10^, ^11^ Therefore, the needs of culturally responsive cancer information reference materials for health workers, key cancer messages for communities, and an integrated health services plan at the district PHC facilities to infuse primary prevention and early detection of cancer into the primary healthcare package remain unmet. Consequentially, the communities in the catchment areas of these health facilities find difficulties in accessing appropriate cancer prevention and early detection interventions.^7^

Moreover, in absence or limited availability of screening equipment in primary healthcare facilities, training and supporting the frontline health workers to create awareness on ways of reducing exposure to cancer risk factors, promoting vaccination against human papillomavirus and hepatitis B virus, and recognizing early warning cancer symptoms and signs (early diagnosis strategy) could reduce the prevalence of exposure to cancer risk factors and facilitate early detection. Of the two early detection strategies of cancer (screening and early diagnosis), early diagnosis is affordable than screening in low‐income countries when targeting the entire population.

Nonetheless, low‐income countries need to implement low‐cost options for primary prevention and screening strategies that should be matched with access to acceptable standards of care within the existing decentralized health services delivery model.^12^ Concerted efforts at all levels are required to counter the premature cancer deaths which is largely linked to late detection^13^ and has become one of the leading causes of premature deaths in low‐income countries among all causes of deaths.^14^, ^15^ Partnering with local community leaders and district primary healthcare managers in mobilization and building trust in the program could leverage program coverage.^16^

Nevertheless, building the capacity of primary healthcare workers with key cancer knowledge, skills and information materials could address some of the difficulties faced by communities in accessing cancer prevention and early detection services. This is consistent with the global concept of universal health coverage. The recent implementation science evidence from Peru based on a “step‐wise five components” model of breast cancer early detection provided a feasible and promising results,^17^ wort replicating in a low‐income setting like Uganda to improve access to early detection.

Overall, to respond to these cancer control needs, in this intervention, we aimed to contribute to reducing the unmet needs of cancer prevention and early detection services in Uganda. To contribute toward reducing these unmet needs, we specifically aimed at building the capacity of PHC workers through engaging district leaders and healthcare managers through regional consultative meetings, development of cancer education, information and communication (IEC) materials and training the district PHC workers on cancer prevention, early detection, referral to tertiary healthcare facilities, community‐based care, how to communicate basic health information on cancer with patients and community members; providing cancer awareness and health education services in the communities, health facilities, schools and other settings.

## 2 METHODS

This capacity building efforts to integrate primary prevention and early detection of cancer into the existing PHC facilities involved stakeholders’ consultative meetings, development and distribution of cancer reference IEC materials for health workers and communities, training of district PHC workers and follow‐up. We targeted all the 122 districts in Uganda based on number of districts in Uganda as in 2017. The participants were sampled purposively with specified inclusion criteria. Invitation letters were emailed to the district health officers to mobilize the specified category of district leaders who were willing to participate in the regional level consultation meetings. Invitation letters were also emailed to the district health officer to identify four health workers who were willing to be trained.

### 2.1 Stakeholders’ regional consultative meetings

**FIGURE 1:**
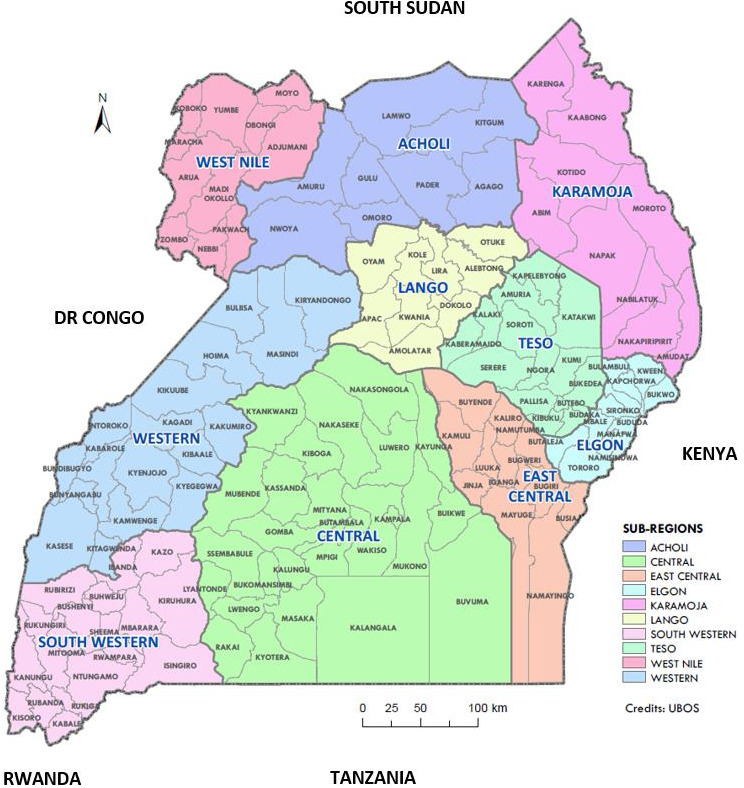
Map of Uganda showing the regions, subregions, and districts where the district stakeholders’ meetings and training of PHC workers were conducted. Adapted from: Uganda Bureau of Statistics (UBOS). In this map, Western region = Western and South Western, Northern = Acholi, West Nile and Lango, Eastern region = East‐central, Elgon, Teso and Karamoja. During the capacity building activities, Acholi, Lango, and Karamoja were zoned as Northern region and North‐western districts zoned with West Nile.

In 2017, we mobilized and conducted one‐day 6 regional cancer control stakeholders’ consultative meetings with representation from 118 out of the planned 122 districts (Table 1 and Figure 1). A maximum of 4 participants per district were invited. The stakeholders included were the District health officers (DHOs)‐head of district health services, Chief administrative officers (CAOs)‐head of district technical services, Chairperson district local council V (CPLCV)‐political head of the district local government, and member of the civil society organizations (CSOs)‐Non‐governmental health related program implementers and advocates. This was done to get input on what is needed and to advocate for their local leadership support for the inclusion of primary prevention and early detection of cancer in their district health work plans. The narrated view points and recommendations of the stakeholders were captured “as said” through note taking and audio‐recording to inform the subsequent capacity building process and interventions.

**TABLE 1:** Regional cancer control stakeholders’ consultative meetings, 2017, Uganda.

Regions	No. of districts Represented	Meeting centers	Attendance		
			Male	Female	Total
East‐central subregion	16	Jinja	29	07	36
Eastern region	21	Mbale	48	10	58
West Nile and North‐western (Bunyoro) subregions	15	Arua	19	11	30
Western region	22	Mbarara	48	10	58
Central region	21	Kampala	23	19	42
Northern region & Karamoja subregion	23	Gulu	52	07	59
Total	118	6	219	64	283

### 2.2 Development of Cancer IEC materials

We developed print and audiovisual cancer education, information and communication (IEC) materials on prevention, early detection and referral based on the commonest types of cancer in Uganda. We reviewed published literatures including World health organization (WHO) publications to guide the development of these IEC materials. These materials were developed to provide reference information for the health workers and community village health teams (VHTs) with priority on the most common types of cancer in Uganda‐especially cervical, breast, and prostate cancer.

### 2.3 Training of district PHC workers

In 2017 and 2018, a maximum of four PHC workers per district were invited for training. The district health teams included were the assistant district health officer‐in‐charge maternal and child health, medical officer or clinical officer, nursing officer and health educator per district and were selected by the district health management from 122 planned districts. The districts were zoned into 6 regions: West Nile, Central, East‐central, Eastern, Western, and Northern Uganda (Figure 1).

The district PHC workers were intensively trained for five days on cancer primary prevention and early detection including raising cancer awareness in the communities, promotion of human papillomavirus and hepatitis B vaccination, early detection through early diagnosis and screening strategies, referral and community‐based care. Emphasis was put on the most common types of cancer in Uganda: cervical, breast, prostate, Kaposi sarcoma, esophageal, liver, lymphoma, leukemia, colo‐rectal, and childhood cancers. In cancer screening pilot districts in East‐central subregion, 46 health workers from 10 health facilities were further intensively trained for 10 days and equipped to provide routine cancer screening for the most common types of cancer in Uganda.

The early detection training component focused on low‐cost options for low‐ and middle‐income countries. For cervical screening, we emphasized visual inspection with acetic acid (VIA) or Papanicolaou (Pap) smear test and pre‐cancer treatment using cryotherapy or thermocoagulation based on “see and treat” or “see and see and treat” approaches. VIA screening is still the most feasible option in low‐income countries and could still be conducive to the future introduction of HPV screening.^18^ Prostate screening methods were based on prostate‐specific antigen (PSA) blood test and digital rectal examination (DRE) technique for most at risk men.^19^, ^20^ The affordable option for breast screening at district PHC facilities in Uganda was clinical breast examination (CBE) and demonstrating to women self‐breast examination (SBE) technique as part of early diagnosis strategy—not screening. Clients with abnormal or suspicious findings in their breast were to be recommended for or referred for breast ultrasound scan or mammography where applicable. However, it is crucial to reiterate that mammography screening option is complex and resource‐intensive, thus may not be feasible in health‐resources constrained countries.^17^, ^18^, ^21^ Mammography is also reported as being less sensitive in young African women and other women with dense breast tissue.^3^ For all types of cancer amenable by early detection, we focused on public health education on early warning cancer symptoms and signs to facilitate seeking early diagnosis and treatment.

During the training, in early detection of cancer we emphasized the application of a low‐cost options of raising cancer awareness and early detection in the communities from lowest level community health facilities through the health service delivery hierarchies, similar to the recent “step‐wise five components” model that was tested in Peru in the context of low‐ and middle‐income countries.^17^ This involves 1‐health education to raise awareness on risk factors, early warning symptoms and signs, and need for screening; 2‐clinical or laboratory‐based screening in a primary healthcare setting; 3‐triage for imaging or laboratory test for detected or suspected lesions or other abnormality during a clinical examination; and 4‐referral of clients with positive or cancer suspicious findings for further management in tertiary hospitals.^17^

We used interactive lectures, demonstration and returned demonstration, role plays and group discussion as the main teaching and learning methods. We assessed learning progress through pre‐ and post‐tests based on a set of questions that covered the entire training content. We also conducted a follow‐up in the districts after six months to approximately one year after the training—as part of process evaluation.

## 3 RESULTS

### 3.1 Stakeholders’ regional consultative meetings

A total of 283 stakeholders from 118 out of the planned 122 districts (Table 1) were engaged in regional consultative meetings in six regional centers. The stakeholders resolved that there was a huge demand for cancer prevention and early detection system strengthening at district primary health facility levels. “When you check our district health centers’ workplans, there is no component of cancer prevention or early detection in them” (Participant East‐central subregion). “The health talks from the district health workers, private health centers and even non‐governmental organizations are on malaria, HIV/AIDS, use of modern contraceptives, and sanitation. Nothing on cancer, yet many people are suffering and dying from cancer” (Participant, West Nile subregion).

District primary healthcare workers being the frontline health workers should be equipped with adequate key cancer information that is suitable for the public and should be provided with IEC materials. “Our health workers need to be trained on key cancer information that the public need to know especially on the risk factors, signs and symptoms, and referral mechanisms for suspected cancer” (Participant, Eastern region).

“PHC workers need simplified guides and booklets on the commonest cancer in Uganda, for example cancer of the cervix, breast and prostate" (Participant, Central region). "These materials can act as reference materials for them” (Participant, Central region).

The importance of including community cancer awareness, early detection, and appropriate referrals in district health work plan and budgets was also stressed. “For sustainability, district health facilities should incorporate cancer awareness and early detection in their annual work plans” (Participant, East‐central subregion). “Government should provide funding for cancer awareness and early detection in the district health budget allocation because the current funding level is over‐stressed by other health priorities other than cancer control activities” (Participant, Western region). “Without more funding, it may not be feasible for the district to add additional priority areas” (Participant, Northern region).

It was envisaged that the high unmet community‐needs for cancer information and part of early detection would be reduced if PHC facilities were strengthened in the context of feasible cancer control interventions.

### 3.2 Development of Cancer IEC materials

**FIGURE 2:**
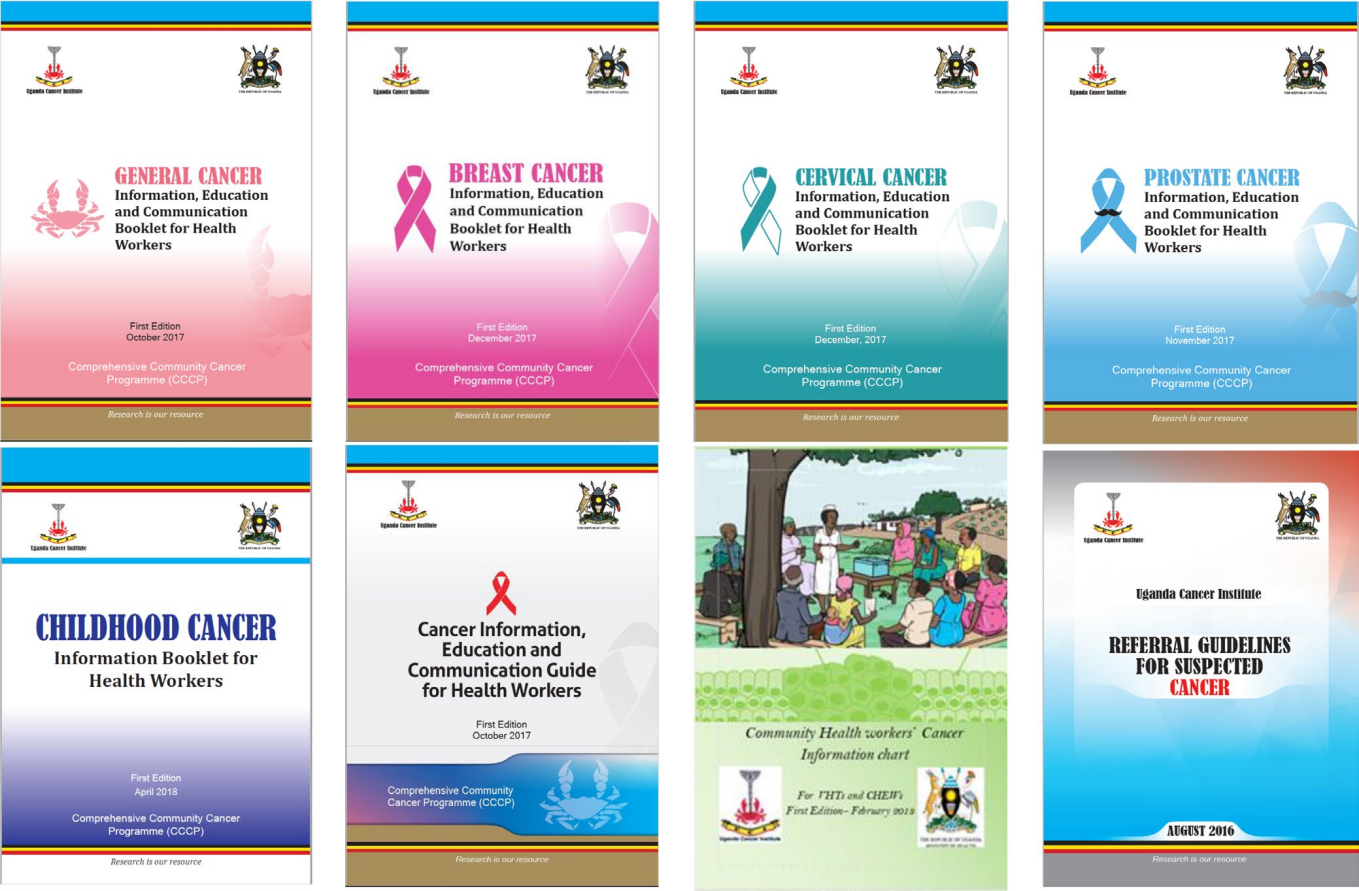
Cancer information, education, and communication materials for primary healthcare workers and community village health teams.

We developed and produced 32,800 cancer IEC materials; breast, cervical, prostate childhood and general cancer information booklets; health education guide and referral guidelines for suspected cases of cancer for health workers and community cancer information flipcharts for VHTs (Table 2). These materials were distributed to 122 districts and fourteen public regional referral hospitals (RRHs), two private‐not‐for‐profit hospitals and one health training institution (HTI) were also allocated the IEC materials (Figure 2 and Table 2). These booklets were also uploaded in the UCI’s website, accessible at https://www.uci.or.ug/download/publications.

**TABLE 2:** Distribution of cancer information booklets and guidelines to district primary healthcare workers in Uganda, 2018.

SN	Region	No. of districts & RRHs	Booklets & Guidelines									
			1‐Breast cancer IEC booklets for health workers	2‐Cervical cancer IEC booklets for health workers	3‐Prostate cancer IEC booklets for health workers	4‐Childhood cancer Information booklets for health workers	5‐Referral guidelines for suspected cancer	6‐Cancer health education & communication Guide for health workers	7‐General cancer IEC booklets for health workers	8‐Cancer treatment guidelines	9‐Community Cancer information flipchart	Total
1	East‐central	12	420	420	420	420	144	120	120	0	660	2724
2	Eastern	29	1015	1015	1015	1015	348	290	290	0	1,595	6583
3	West Nile	09	315	315	315	315	108	90	90	0	495	2043
4	Northern	16	560	560	560	560	192	160	160	0	880	3632
5	Western	31	1,085	1,085	1,085	1,085	372	310	310	0	1,705	7037
6	Central	25	875	875	875	875	300	250	250	0	1,375	5675
	District‐total	122	4,270	4,270	4,270	4,270	1,464	1,220	1,220	0	6,710	27694
7	RRHs &HTI	17	730	730	730	730	536	280	280	300	790	5106
	Total	139	5000	5000	5000	5000	2000	1500	1500	300	7500	32,800

**FIGURE 3:**
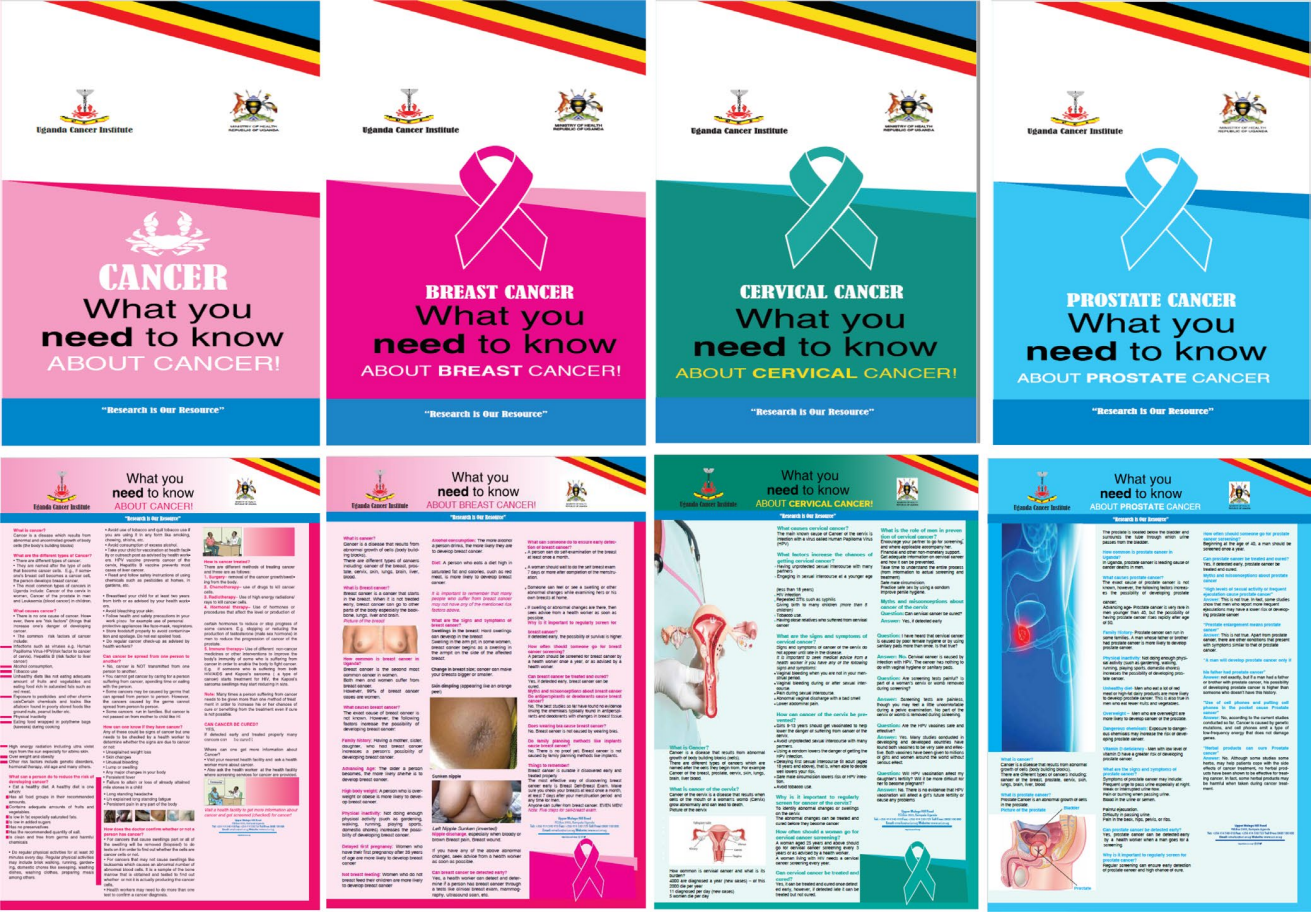
Cancer information brochures and posters for public cancer awareness.

**FIGURE 4:**
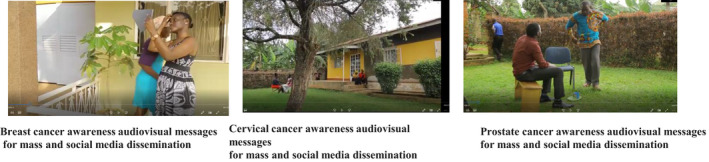
Breast, cervical, and prostate health awareness audiovisual messages in five most common languages in Uganda.

We also developed brochures and posters (Figure 3) for the public and audiovisual messages on breast, cervical, and prostate cancer for mass and social media (Figure 4). These clips were translated from English to four other commonest languages in Uganda: Luganda, Luo, Runyankole‐Rukiga, and Ateso, and disseminated to the public via social media platforms like Facebook and WhatsApp, including UCI’ Facebook page.

The key messages in each of the audiovisual clips include what is cancer, the risk factors of the specific type of cancer, preventive measures, screening and early diagnosis, access to care, where to seek help and reassurance that prevention is better than waiting for cure that could be difficult to achieve when cancer has spread, and early detection saves a life.

### 3.3 Training of district PHC workers

A total of 488 health workers (482 district health workers and six cancer survivors’ volunteers) from 118 out of 122 planned districts were trained (Table 3). Cancer survivors’ volunteers were included in this training to equip them basic cancer key messages to support raising cancer awareness and demonstrating the evidence of cancer survival, and benefit of early detection in saving life through sharing lived experience in the communities. During the training, each district health team were advised on the need to identify a focal point person in their district on matters concerning primary prevention and early detection of cancer. Forty‐six health workers in the pilot East‐central subregion (Figure 1) were further trained in cervical, breast, and prostate cancer screening techniques including health education for early diagnosis‐this is anticipated to be replicated in other regions in phases. The distribution of pre‐ and post‐test training evaluation indicated a good improvement in the level of cancer knowledge among the trainees (Table 4).

**TABLE 3:** Number of district primary healthcare workers per region trained on primary prevention and early detection of cancer, 2017‐2018, Uganda.

Regions	No. of districts	Regional training centers	Number trained		
			Male	Female	Total
East‐central subregion	16	Jinja	27	36	63
Eastern region	21	Mbale	42	42	84
Central region	21	Kampala	31	49	80
West Nile & North‐western (Bunyoro subregions	15	Arua	22	44	66
Northern region & Karamoja subregion	23	Gulu	45	47	92
Western region	22	Mbarara	57	40	97
Cancer survivors’ volunteers	—	—	0	6	6
Total	118	06	224	258	488

**TABLE 4:** A comparison of the participants’ pre‐ and post‐training evaluation, 2017‐2018, Uganda

Overall & regional performance score	Pre‐ and post‐test percentage (%) scores.	Proportion of participants in each category of pretest score n (%)	Proportion of participants in each category of post‐test score n (%)
Overall test scores	*70‐100*	79 (16)	371 (77)
	*50‐69*	175 (36)	100 (21)
	*0‐49*	228 (48)	11 (2)
	**Total**	**482**	**482**
East‐central	*70‐100*	*8 (13)*	*58 (92)*
	*50‐69*	*28 (44)*	*5 (8)*
	*0‐ 49*	*27 (43)*	*00 (0)*
	**Sub‐total**	**63**	**63**
Eastern	*70‐100*	*22 (26)*	*68 (81)*
	*50‐69*	*30 (36)*	*15 (18)*
	*0‐49*	*32 (38)*	*01 (1)*
	**Sub‐total**	**84**	**84**
Central	*70‐100*	*23 (29)*	*71 (89)*
	*50‐69*	*40 (50)*	*9 (11)*
	*0‐ 49*	*17 (21)*	*00 (0)*
	**Sub‐total**	**80**	**80**
West Nile	70‐100	11 (17)	54 (82)
	50‐69	21 (32)	11 (17)
	0‐ 49	34 (51)	1 (1)
	**Sub‐total**	**66**	**66**
Northern	*70‐100*	*09 (10)*	*59 (64)*
	*50‐69*	*32 (35)*	*30 (33)*
	*0‐ 49*	*51 (51)*	*03 (3)*
	**Sub‐total**	**92**	**92**
Western	*70‐100*	*06 (6)*	*61 (63)*
	*50‐69*	*24 (25)*	*30 (31)*
	*0‐ 49*	*67 (69)*	*06 (6)*
	**Sub‐total**	**97**	**97**
Total		**488** (482+6 cancer survivors)	**488** (482+6 cancer survivors)

### 3.4 Follow‐up and agreed actions

Approximately six months to one year after the training, district health officers were either met in their districts or contacted through phone calls to discuss the actions taken, challenges and proposed actions following training of the selected health workers. By June 2019, 75% of the districts said they had conducted at least one; continuous medical education (CMEs) on basic cancer information to the health facility staff that were not trained; health facility‐based cancer education to patients; community cancer awareness outreaches and radio talk shows. The sources of radio talk shows airtime were mainly from the resident district commissioners (RDCs). In Uganda, the RDCs are usually allocated airtime for communicating crosscutting government programs to the communities through the local radio stations. The pilot sites for cancer screening in East‐central subregion reported initiation of routine cancer health education sessions and screening for cervical and breast cancer in their health facilities.

The main challenge met during the implementation of this capacity building efforts was limited funds to train adequate number and multi‐disciplinary cadres of district PHC workers. We were able to train only four health workers in each of the 118 districts out of 122 districts in Uganda in 2017. The four districts that missed the training received all the IEC materials and they were scheduled for subsequent training based on availability of funds. The challenges reported by the districts were inadequate funds and cancer screening supplies especially vaginal speculums for cervical screening based on VIA. The agreed actions included the need for cancer awareness and early detection work plan with allocated funds in the district health work plan starting 2020/2021 financial year (FY). Integration of cancer awareness messages in routine district health services like HIV/AIDS, nutrition and environmental health in health facilities, schools and communities and collaboration with community‐based organizations in their districts were also emphasized.

## 4 DISCUSSION

To contribute toward reducing the unmet needs for cancer control services in Uganda, we engaged district leaders and healthcare managers through regional consultative meetings, developed and distributed cancer education, information and communication materials to the districts and regional hospitals, and trained the district PHC workers on cancer prevention, low‐cost early detection options, referral to tertiary healthcare facilities, community‐based care, how to communicate basic health information on cancer with patients and community members; conducting cancer awareness in the communities, health facilities, schools and other settings.

Studies have attributed the low level of cancer awareness and detection of cancer in advanced stages to limited knowledge on the benefits of early detection and timely cancer management^6^, ^7^ and scarcity of adequate health facilities with the capacity to provide cancer information, screening and diagnostic services.^8^, ^9^, ^10^, ^11^ Despite cancer control interventions being resource‐intensive, low‐income countries could implement the low‐cost options for primary prevention and early detection that should be matched with access to acceptable standards of care.^12^ Therefore, we anticipate that this capacity building if sustained could bring services nearer to the people, encourage adoption of preventive health behaviors, affordable early detection services and improve follow‐up and care in the community.

The adoption of new preventive and screening options that are easy‐to‐use, highly accurate, low‐cost and that could be provided to the clients at the point of care is feasible and effective in addressing the difficulty in accessing cancer prevention and early detection services.^22^ In low‐resource areas, evidence suggests that the adoption of an affordable model for cancer prevention and early detection to address such limitation is feasible.^22^ Therefore, it could be feasible if the government and civil society organizations (CSOs) support the sustenance of this integration into the district PHC facilities in Uganda. This is expected to contribute in reducing the unmet needs of cancer prevention and early detection services in Uganda.

To support the PHC workers, culturally appropriate reference cancer information, education and communication (IEC) materials are necessary to act as guides for cancer awareness in specific communities and providing early detection and referral services in their health facilities.^23^ Evidence suggests that printed culturally responsive reference booklets for community health workers could help health workers in creating cancer awareness in specific communities.^23^ The IEC materials that were developed through this capacity building efforts are also anticipated to support health facility‐based training of additional health workers including the community‐based health volunteers “the Village health teams” (VHTs) to link the community with the professional health workers through dissemination of key cancer messages in their communities.^24^

The alignment of primary prevention and early detection of cancer in a “step‐wise approach” for low‐resource settings was demonstrated to be feasible and effective in a six years of breast cancer early detection implementation research in Peru.^17^ During this capacity building process, we emphasized an approach similar to the Peruvian five components’ model: 1‐health education to raise awareness on risk factors, early warning symptoms and signs, and need for screening; 2‐clinical or laboratory‐based screening in a primary healthcare setting; 3‐triage for imaging or laboratory test for detected or suspected lesions or other abnormality during a clinical examination; 4‐referral of clients with positive screen findings for further management or referring cases with cancer suspicious lesions for biopsy sampling in nearby by hospitals; and 5‐patient referral and navigation to tertiary cancer care hospital for timely management.^17^ We expect that this approach could also be feasible in Ugandan settings and contribute in reducing the extent of the current cancer control health disparities in Uganda, in particular the difficulties experienced by communities in accessing cancer prevention and early detection services.^22^ Nevertheless, concerted efforts of various stakeholders are required to counter the catastrophic economic and social burden of cancer.^13^

Also, the leverage from community‐based assets should be considered in supporting cancer control interventions that are applicable at PHC facilities and communities. It is possible to tap from the community‐based assets to support primary prevention and early detection of cancer in the communities where applicable.^25^ Taking advantage of community social centers, places of worships, schools, women (mother) unions, social gatherings, community radios in public markets and urban centers to disseminate key cancer messages to the community is feasible. For example, some district health workers reported conducting cancer awareness talk shows using free airtime provided by the resident district commissioners.

Moreover, despite the relatively high cost of screening for the most common types of cancer like cervical and breast cancer when targeting the entire population at risk, screening at least once in a person’s lifetime and public health education on early symptoms and signs could prompt individuals to seek diagnosis early enough before cancer has spread. Therefore, both screening and early diagnosis strategies are important components of early detection. Apart from cancer of the cervix, breast, colon, rectum and oral cavity, the other types of cancer do not fully meet the criteria for instituting national screening program; however, most of them can be down‐staged through early diagnosis.

Despite funding limitation, implementation of such approaches to build the capacity of health workers to adapt to the competing burden of both communicable diseases such as HIV, Malaria and Tuberculosis and noncommunicable diseases like cancer could improve the readiness of the health system to respond to the societal health services demand in low‐income settings. This is envisaged to contribute to improving cancer awareness and downstaging—reducing the proportion of patients who present with late‐stage cancer through early detection program. Irrespective of the country’s income status, primary prevention and early detection of cancer could be implemented at primary healthcare facilities, though to a varying extent. However, the role of national, regional, and district political leaders and healthcare managers are crucial in making such cancer control interventions to work. Political will and commitment in PHC activities are cornerstones for the success of any primary care program.^26^ Recent implementation research on the prevention of cervical cancer in rural areas of Zambia re‐affirm that working with and through community leaders such as traditional chiefs to increase access to cancer prevention could yield more coverage.^16^ Thus, consultation and inclusion of district technical and political leaders who are the communities’ gate‐keepers in the decentralization and integration of cancer prevention and early detection services is paramount for its successful implementation.

Nevertheless, there is a caveat to take note of. These interventions could potentially create a situation where the demand for both early detection and cancer management (diagnosis, treatment and follow‐up) services in the country exceeds the supply capacity to address these needs. Studies reported that financial and logistical constraints,^21^ limited availability of confirmatory diagnostic pathology, specialized surgical, chemotherapy, and radiotherapy services and specialized oncology health workers could greatly affect the supply side of cancer control services.^27^, ^28^, ^29^, ^30^ Moreover, after detection or suspicion for cancer at PHC facility, the demand side for cancer management could also be influenced by travel, accommodation and other out‐of‐pocket expenditures when seeking further specialized care at regional and national hospitals that are usually located in urban centers far away from the rural residents.^31^, ^32^, ^33^, ^34^, ^35^ It is therefore necessary for the tertiary levels of care (regional and national) to get ready in case of surge in demand for cancer control services; otherwise, the facilities would be overwhelmed.

Thus, the participation and support of the national, regional and district health leadership and community leaders with the training of district primary healthcare workers to build their capacity in cancer prevention and early detection is critical for establishment and implementation of cancer prevention and early detection strategies in the district PHC facilities.^35^, ^36^, ^37^

## 5 CONCLUSIONS

In Uganda, the unmet health needs of cancer control services along the various continuum of cancer control are enormous. However, building the capacity of primary healthcare workers through consultative engagement, development of reference cancer IEC materials, training the district PHC workers on cancer prevention and low‐cost early detection options to integrate primary prevention and early detection of cancer into the existing primary healthcare services are crucial. This could bring services nearer to the people, encourage adoption of preventive health behaviors, early detection and improve follow‐up and care in the community. This may contribute in reducing the unmet needs of cancer prevention and early detection services in Uganda.

## 6 COMPETING INTERESTS

The authors have declared that no competing interests exist.

## 7 CONSENT FOR PUBLICATION

Not applicable.

## Authors' contributions

Alfred Jatho: Project design, development of the training and IEC materials, conducting the training, project reports, manuscript drafting. Noleb Mugume Mugisha: Project design, project management, development of the training and IEC materials, conducting the training, project reports, manuscript review. James Kafeero: Development of the training and IEC materials, conducting the training, project reports, manuscript review. George Holoya: Development of the training and IEC materials, conducting the training, project reports, manuscript review. Fred Okuku: Development of the training and IEC materials, conducting the training, project reports, manuscript review. Nixon Niyonzima: Project management, development of the training and IEC materials, conducting the training, manuscript review. Jackson Orem: Project management, review of IEC materials and manuscript.

## Funding information

The stakeholders’ consultations, development of cancer information materials, and training of district primary healthcare workers were supported by the African Development Bank (AfDB) “East Africa Centre of Excellence for Oncology project” at the Uganda Cancer Institute.

## Ethical statement

Not applicable.

## Data Availability

All relevant data are within this report.
